# Photon-Counting Computed Tomography Enables Image-Guided Navigation of the Paranasal Sinuses at Ultra-Low Radiation Dose: An Initial Cadaveric Study

**DOI:** 10.3390/diagnostics16203279

**Published:** 2026-10-09

**Authors:** Friederike Bärhold, Tobias Albrecht, Bernhard Hirt, Thore Schade-Mann, Agnes Mühle, Johannes M. Schneider, Saif Afat, Andreas Brendlin, Benjamin P. Ernst, Leon Guchlerner, Sven Becker

**Affiliations:** 1 Department of Otorhinolaryngology, Head and Neck Surgery, University Hospital Tübingen, 72076 Tübingen, Germany; 2 Department of Clinical Anatomy and Cell Analysis, Eberhard Karls University, 72076 Tübingen, Germany; 3 Department of Radiology, University Hospital Tübingen, 72076 Tübingen, Germany; 4 Department of Otorhinolaryngology, Head and Neck Surgery, University Hospital Frankfurt, Goethe University, 60596 Frankfurt am Main, Germany

**Keywords:** radiation dose, photon counting computed tomography, image-guided navigation, paranasal sinuses, endoscopic sinus surgery

## Abstract

**Background/Objectives**: Photon-counting computed tomography (PCCT) enables substantial radiation dose reduction while preserving high spatial resolution. Its potential for image-guided navigation in endoscopic sinus surgery (ESS), particularly at ultra-low dose levels, remains insufficiently explored. This study aimed to evaluate the feasibility of PCCT for four different surgical navigation systems at progressively reduced radiation doses. **Methods**: PCCT datasets of the paranasal sinuses were acquired from two cadaveric specimens using a standard protocol followed by stepwise dose reductions down to 5% of the reference dose. The datasets were used for image-guided navigation with four systems (one optical, three electromagnetic). Registration success, navigation accuracy, and user satisfaction (visual analog scale) were assessed at 100% and 5% dose levels using linear mixed-effects models. **Results**: Registration was successful for all navigation systems at every dose level. Mean navigation accuracy remained stable despite dose reduction (2.22 ± 1.16 mm at 100% vs. 1.93 ± 1.05 mm at 5% radiation dose; *p* = 0.364). User satisfaction regarding navigation was consistently high (mean 8.8 ± 0.75) and showed no significant differences between dose levels. **Conclusions**: PCCT enabled image-guided navigation even at ultra-low radiation doses down to 5% of standard exposure, without compromising navigation accuracy in this initial study. These findings highlight the potential of PCCT to substantially reduce radiation burden while maintaining clinical performance. Further in-human studies are needed.

## 1. Introduction

Otorhinolaryngology involves the assessment and treatment of anatomically complex regions characterized by narrow spaces and limited accessibility. While clinical examination remains fundamental, key structures such as the middle ear and paranasal sinuses are not fully accessible by non-invasive means. Consequently, computed tomography (CT) has become an essential diagnostic tool, particularly for evaluating sinonasal anatomy and pathology as well as for preoperative planning [1,2]. CT also supports the assessment of disease extent, the detection of anatomical variants, and the identification of bony remodeling or erosion that may influence therapeutic decision-making [3]. This is especially relevant when endoscopic visualization is limited by mucosal swelling, retained secretions, or postoperative changes.

In endoscopic sinus surgery (ESS), precise anatomical orientation is critical to avoid complications involving adjacent structures such as the skull base, orbit, optic nerve and internal carotid artery [4,5]. CT imaging plays a central role in surgical planning and is indispensable for image-guided surgery (IGS), especially in complex cases such as revision procedures or oncologic interventions with potential skull base involvement [6,7]. CT-based navigation systems, using optical or electromagnetic tracking, rely on accurate preoperative imaging to ensure intraoperative precision [8,9,10]. In revision surgery, altered anatomy and scar tissue may further obscure normal landmarks, increasing the value of preoperative imaging and navigation support. However, the accuracy of navigation is influenced not only by the tracking modality and registration process, but also by the spatial resolution and overall fidelity of the imaging dataset [11].

Photon-counting computed tomography (PCCT) is an emerging imaging technology that differs fundamentally from conventional energy-integrating detector (EID) CT [12,13]. By directly converting X-ray photons into electrical signals, PCCT enables higher spatial resolution and improved depiction of delicate anatomical structures [14,15]. Previous studies have demonstrated that PCCT provides excellent image quality for imaging of the paranasal sinuses and skull base, even at reduced radiation dose levels [16]. Initial investigations in cadaveric and in vivo settings suggested that PCCT of the paranasal sinuses outperforms conventional CT techniques in terms of image quality while enabling dose reduction [17,18]. This was confirmed by a larger in vivo study comparing ultra-low PCCT with cone-beam CT (CBCT) [19]. For sinonasal imaging, this combination is particularly relevant because thin bony partitions, small foramina, and neurovascular canals must remain clearly visible despite dose reduction. In addition, many patients with chronic rhinosinusitis or postoperative disease require repeated imaging over time, making radiation optimization an important consideration. Despite these promising findings, its potential for image-guided navigation in ESS, particularly at ultra-low dose levels, remains insufficiently explored.

A related study from Ernst et al. demonstrated that PCCT provides excellent accuracy of anatomical landmarks in comparison with dual-energy dual-source CT (DECT), and spectral detector CT (SDCT) in image-guided surgery, along with superior image quality of the paranasal sinuses in low-mA scans and substantially reduced radiation exposure [20]. Building on these findings, the present study investigates whether PCCT datasets acquired at progressively reduced radiation dose levels remain suitable for use across four different surgical navigation systems. In doing so, the study aims to clarify whether ultra-low-dose PCCT can be integrated into routine preoperative workflows without compromising navigational reliability.

## 2. Materials and Methods

### 2.1. Study Design

In this prospective single-center study, PCCT images were acquired from two body donors and subsequently used for navigation with four different CT-guided navigation systems. Navigation accuracy and user satisfaction were assessed across standard and reduced radiation dose protocols.

Ethical approval was obtained (project number 563/2024BO2). All body donors had provided informed written consent prior to death.

### 2.2. Imaging Protocol

Two body donors, one male (85 y at death), one female (95 y at death), without prior sinus surgery, fixed using an alcohol-based solution within 72 h after death developed by the Department of Anatomy, University of Tübingen, were examined using a NAEOTOM Alpha PCCT scanner (Siemens Healthcare GmbH, Erlangen, Germany). Images were acquired 32 and 35 days after death, respectively. Standard imaging was performed at Sn100 kV and IQ level 24, corresponding to the routine sinus CT protocol at the Department of Radiology, University Hospital of Tübingen. Subsequently, tube current and IQ-level were reduced stepwise to 50%, 25%, 10%, and 5% of the initial settings, resulting in proportional reductions in radiation dose. The derived radiation exposure parameters for the two body donors using the reference dose protocol are summarized in Table 1: The computed tomography dose index volume (CTDI_vol) measured 3.23 mGy and 3.05 mGy for specimens 1 and 2, respectively, corresponding to a mean CTDI_vol of 3.14 mGy. The scan length was 15.7 and 19.8 cm, respectively. The associated dose-length product (DLP) values were 50.5 mGy*cm and 60.3 mGy*cm, respectively, resulting in a mean DLP of 55.4 mGy*cm. The corresponding effective radiation doses across both body donors are summarized in Table 2: Mean CTDI_vol values decreased from 3.14 mGy at the 100% reference dose level to 1.55 mGy, 0.78 mGy, 0.26 mGy, and 0.13 mGy at the 50%, 25%, 10%, and 5% dose levels, respectively. Likewise, the mean dose-length product (DLP) was reduced from 55.4 mGy*cm at the reference protocol to 27.5 mGy*cm, 13.8 mGy*cm, 4.5 mGy*cm, and 2.2 mGy*cm. The estimated effective radiation dose decreased proportionally from 0.83 mSv at the standard protocol to 0.41 mSv, 0.21 mSv, 0.07 mSv, and 0.03 mSv, respectively. All image datasets were reconstructed using identical reconstruction settings to ensure comparability between dose levels and to avoid confounding effects related to image post-processing. Particular attention was paid to maintaining adequate visualization of surgically relevant anatomical structures. The effective dose E was estimated based on tissue weighting factors $w_{T}$ and equivalent organ doses $H_{T}$ according to ICRP Publication 103 (2007) [21].

### 2.3. Navigation Device Setup and Endoscopy

The four different CT-guided navigation devices Kick^®^ Cranial/ENT Navigation Software ENT version 3.1.5, Brainlab AG, Munich, Germany; Stryker ENT Navigation System, Scopis ENT Software with TGS, REF 8000-020-002, version 3.4, Stryker Corporation, Kalamazoo, MI, USA; STEALTHSTATION™, Medtronic, Minneapolis, MN, USA; and Fiagon ENT Navigation System, software version 5114.30, Fiagon GmbH, Hennigsdorf, Germany were used to calibrate and navigate the acquired 100% and 5% dose images. Initial calibration was performed using full-dose (100%) datasets across the four clinically approved navigation systems. Following successful registration, the 100% dose images were used for navigation followed by 5% and subsequent increase in reduced-dose datasets (10%, 25%, and 50%).

Electromagnetic systems were calibrated independently by a trained company representative and an experienced sinus surgeon. The optical system was calibrated by two sinus surgeons who had previously completed manufacturer training. A reference marker was attached to the forehead of each specimen. Calibration was performed using point-based (Brainlab, Fiagon) calibration or a combination of point-based and surface calibration (Medtronic, Stryker). To minimize technical variability, all navigation procedures were performed under standardized laboratory conditions with unchanged specimen positioning throughout the experiments. Calibration procedures were repeated whenever registration inaccuracies or technical inconsistencies were suspected. Subsequently, four sinus surgeons navigated predefined anatomical landmarks (nasion, nasal spine, bilateral canthus, bilateral middle turbinate heads, and posterior nasopharyngeal wall) using rigid 0° or 30° endoscopes and calibrated pointers. For capacity reasons, two specimen were navigated with the Fiagon and Stryker electromagnetic devices, and one specimen with Brainlab optical device and Medtronic electromagnetic device, respectively. Navigation accuracy was documented by capturing screenshots during pointer placement at each landmark. Measurements were obtained either using integrated device rulers or via calibrated overlay with the original CT datasets.

### 2.4. Analysis

Navigation accuracy was defined as the distance (mm) between the pointer position and the corresponding anatomical landmark in the CT dataset. User satisfaction with the calibration process was assessed using a visual analog scale (VAS; 0 = dissatisfied, 10 = fully satisfied), comparing full-dose and ultra-low-dose (5%) datasets. Four experienced sinus surgeons performed all evaluations. Potential influences of navigation device, examiner, and calibration method (point or combined point and surface) were analyzed.

Measurement discrepancy (mm) was analyzed using means and standard deviations. In order to assess differences between navigation accuracy at 100% and 5% radiation dose a linear mixed-effects model was applied with radiation dose and specimen as fixed effects, while examiner was included as the random-effect grouping variable and navigation system as an additional random-effect variance component.

User satisfaction was analyzed descriptively (mean ± standard deviation). Additionally, analysis of variance (ANOVA) was performed to assess the influence of radiation dose and navigation device on user satisfaction. All statistical analyses were conducted using Python, version 3.10.20, Software Foundation (2016), with the additional packages pandas (version 2.3.3) and statsmodels (version 0.15.0) [22,23].

## 3. Results

### 3.1. Radiation Dose

**Figure 1:**
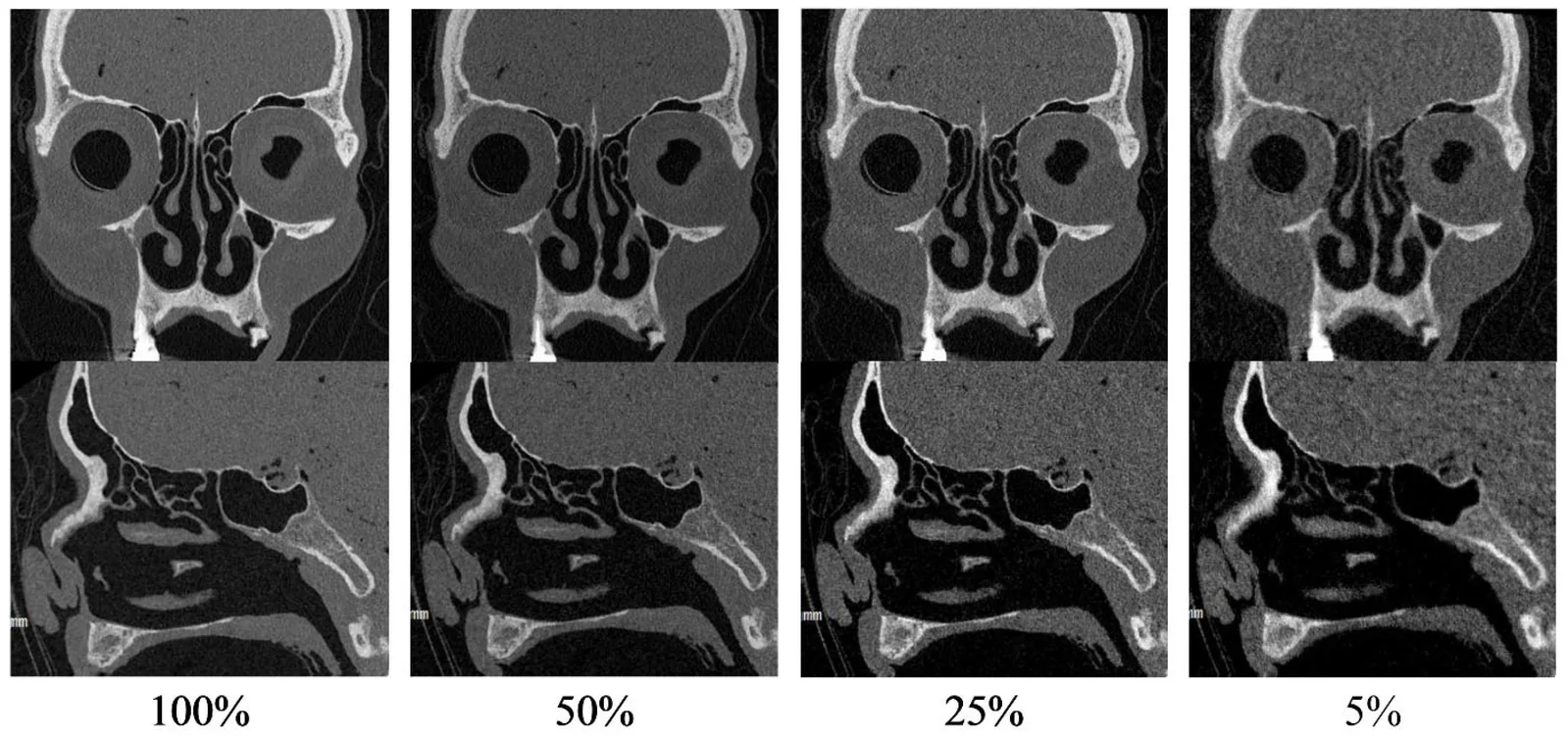
Representative coronal (**top**) and sagittal (**bottom**) PCCT images of the paranasal sinuses from a single body donor acquired at progressively reduced radiation dose levels (100%, 50%, 25%, and 5% of the reference dose, from left to right).

**Figure 2:**
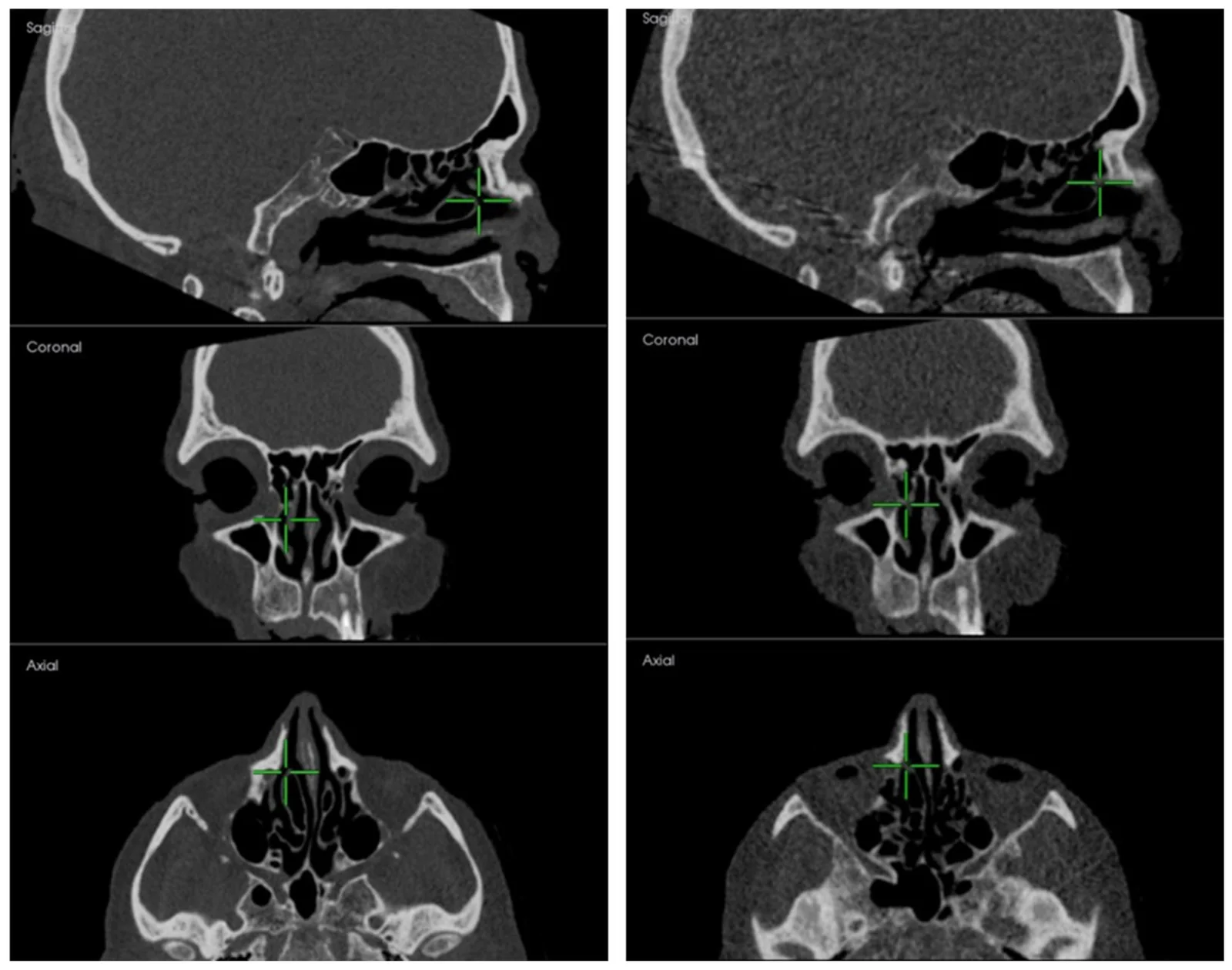
Representative navigation screenshots from one body donor using PCCT datasets. The position of the navigation pointer is indicated by a green crosshair. Full-dose images (100%) are shown on (**left**), and ultra–low-dose images (5% of the reference dose) on (**right**).

At full settings (100%), the mean effective radiation dose was 0.83 mSv. This value decreased to 0.41 mSv at 50% and further to 0.03 mSv at 5% of the initial settings. Representative PCCT images and navigation screenshots at different dose levels are shown in Figure 1 and Figure 2. Compared to 100% dose images, decreased radiation dose led to an increase in image noise especially of the soft tissue (see also Figure 1 and Figure 2).

### 3.2. Navigation Deviation

Calibration was successfully achieved with all navigation systems at every dose level, independent of image resolution or calibration method. Because of successful calibration and similarly accurate navigation at 5% compared to 100% dose level, the intermittent dose steps (10%, 25%, 50%) were not used for navigation. A total of 189 navigation measurements were conducted. The overall mean navigation error was 2.01 ± 2.09 mm. No significant difference in accuracy was observed between full-dose and ultra-low-dose datasets, with mean deviations of 2.22 ± 1.16 mm at 100% and 1.93 ± 1.05 mm at 5% (-*p* = 0.288). Analysis of individual anatomical landmarks revealed the greatest deviation at the posterior nasopharyngeal wall (2.98 ± 2.58 mm), whereas the highest accuracy was observed at the bilateral canthi (1.30 ± 1.77 mm). An overview of navigation deviation across all conditions is provided in Table 3.

Navigation accuracy did not differ significantly between devices (*p* = 0.71), although mean deviations ranged from 1.41 ± 0.94 mm for the most accurate system to 2.74 ± 0.82 mm for the least accurate. Pairwise comparison between navigation devices by Stryker and Fiagon showed inconsistent agreement across landmarks, with overall weak correlation ranging from r = 0.72 (spina nasalis superior) to r = 0.42 (nasopharynx), alongside systematic mean differences of 1–5 mm between systems for several landmarks. Pairwise comparison with Medtronic and Brainlab were based on only 4 shared conditions (versus 8 for Stryker and Fiagon) and are too limited to draw conclusions. The calibration method (point-based or combined point and surface based) had no significant effect on navigation accuracy (*p* = 0.11).

### 3.3. Inter-Rater Reliability

Variations between examiners were observed, with one examiner achieving a mean deviation of 0.65 ± 0.51 mm, while the remaining examiners showed values ranging from 2.05 ± 0.4 mm to 2.2 ± 1.1 mm. Inter-rater reliability could be assessed between two of the examiners only due to a smaller number of conducted measurements by the other examiners. Inter-rater reliability showed good-to-excellent agreement on most landmarks (turbinates, spina nasalis, canthus all r > 0.67), with somewhat weaker (but still moderate) agreement specifically for the nasopharynx (r = 0.56–0.68).

### 3.4. User Satisfaction

A total of 25 individual evaluations on a visual analog scale were given by the surgeons. User satisfaction with calibration was high across all conditions (mean VAS: 8.8 ± 0.8). No significant differences were observed between full-dose and 5% dose datasets (8.7 ± 0.9 vs. 8.8 ± 0.7; *p* = 0.278). Neither navigation device nor calibration method significantly influenced user satisfaction (*p* = 0.13 and *p* = 0.21, respectively).

## 4. Discussion

This proof-of-concept study evaluates the use of ultra-low-dose Photon Counting CT images across different image-guided navigation systems. While prior studies have focused on diagnostic image quality and dose efficiency, the question of whether these advantages translate into reliable intraoperative navigation has remained largely unexplored. Our study addresses this gap by demonstrating that the technical improvements of PCCT are sufficient not only for diagnosis but also for spatially precise navigation in a preliminary cadaveric study. Here, PCCT images could be reduced to radiation doses as low as 5% of the standard exposure without compromising navigation accuracy, which was measured by deviation in mm from predefined anatomical landmarks. Other potentially influencing factors on navigation accuracy were considered in the linear mixed-effects model, including examiner and navigation system. Correcting for these effects, the mean navigation deviation after 95% dose reduction was comparable to the accuracy obtained with full-dose imaging. Across all eight anatomical landmarks, reduction of radiation dose from 100% to 5% was not associated with a statistically significant increase in registration deviation (all *p* ≥ 0.069, except spina nasalis superior, *p* = 0.023, where deviation was, unexpectedly, slightly lower at reduced dose). Overall, these findings support that navigation accuracy is preserved across anatomical landmarks even at substantially reduced radiation dose. Successful registration was achieved with all navigation systems across all dose levels, and user satisfaction remained consistently high without significant differences between dose levels, devices or registration methods. Inter-rater reliability was good-to-excellent for most landmarks, with the nasopharynx demonstrating weaker agreement. The nasopharynx is less accessible, further from calibration points and composed of soft tissue, leading to this effect. Pairwise comparisons of navigation devices suggest limited and inconsistent agreement between navigation systems in this sample, warranting cautious interpretation and further investigation with a larger number of shared measurement conditions.

Previous studies were conducted to assess navigation accuracy and setup of different devices over the last decades. The reported navigation accuracy ranged from 0.1 to 2.3 mm (1.5–2.3 mm [24], 0.1–0.5 mm [25], and 1.29 ± 0.29 mm [26], respectively), with the accuracy of the here-conducted study falling within this range (2.01 ± 2.09 mm). The comparatively broader standard deviation observed in our study may be attributable to the inclusion of multiple navigation systems and anatomical landmarks, as well as the cadaveric setup, which differs from controlled phantom-based measurements often reported in the literature.

Since the clinical introduction of PCCT in 2021 [27], multiple studies have demonstrated its ability to reduce radiation dose while preserving or even improving image quality [15,28,29]. Prior work on temporal bone and sinus imaging has highlighted superior spatial resolution and diagnostic performance at reduced dose levels [17,18]. Rajendran et al. reported superior image quality compared to conventional CT at standard dose [16]. Ernst et al. showed that PCCT allowed a 45% radiation dose reduction while maintaining superior diagnostic image quality compared to DECT and SDCT [20]. Additionally, a single-center retrospective blinded study demonstrated that diagnostic PCCT of the paranasal sinuses outperforms cone beam CT in terms of subjectively rated image quality at substantially lower radiation exposure [19]. Superior image-quality has also been demonstrated for brain and temporal bone amongst others. Building on these findings, the present study examines the application [14,16,30,31] of PCCT to image-guided navigation at ultra-low-dose levels in the paranasal sinuses. The preservation of navigation accuracy at ultra-low-dose levels may be explained by the intrinsic properties of photon-counting detectors, including reduced electronic noise, improved signal-to-noise ratio, and enhanced spatial resolution compared with conventional EID-CT. These characteristics allow sufficient anatomical detail for registration and navigation even when image noise increases at lower dose levels. Despite superiority compared to EID-CT, PCCT is still prone to increased image noise especially at reduced dose levels. Several post-processing techniques have been implemented in clinical practice and studies to account for this problem with increasing use of artificial intelligence (AI). AI-based denoising has been shown to significantly improve low-dose PCCT images both in sinusitis [32] as well as the whole body [33] among others, making even lower doses for diagnostic purposes feasible. No AI-based post-processing was used in the here-presented study but is a promising method also for this purpose.

The strength of this study is the use and comparison of four separate navigation devices from four different companies with a total number of 189 total measurements. The difference in accuracy and user satisfaction did not differ significantly between the devices. However, in our study, accuracy varied markedly between different anatomical landmarks. The nasopharynx demonstrated lowest accuracy (2.73 ± 2.41) whereas the middle turbinate and canthus showed the highest accuracy (1.79 ± 1.95 and 1.86 ± 2.03, respectively). This difference can be explained by calibration geometry and texture of the landmarks. Structures such as the canthus, which are commonly included in calibration, showed higher precision. In contrast, the nasopharynx, being more distant from calibration points and composed of softer tissue, exhibited greater deviation. In addition, image noise at reduced dose levels may disproportionately affect low-contrast soft tissue regions such as the nasopharynx, whereas high-contrast bony structures remain well defined. For intraoperative navigation of the paranasal sinuses, the bony landmarks are decisive for guiding the surgeon. Those proved to be most accurate in our cadaveric study, with their outline being visibly less affected by reduced radiation dose compared to soft tissue.

The ability to perform accurate navigation using only 5% of the standard radiation dose may have potential clinical implications. Radiation exposure remains a critical concern in medical imaging, particularly in accordance with the “As Low As Reasonably Achievable” (ALARA) principle and its current application in head and neck imaging [34]. While previous dose-reduction strategies such as tin filtration and cone beam CT have been explored, these approaches are often limited by reduced image quality [35,36]. PCCT represents a substantial advancement, enabling significant dose reduction without compromising clinical usability. Particularly in children radiation exposure should be avoided due to long-term complications including primarily stochastic effects (cancer, heritable mutations). Low-dose PCCT imaging has the potential to significantly reduce radiation dose in further otorhinolaryngology applications. A recent publication showed that ultra-low dose PCCT images could also be used for the preoperative planning of cochlear implants [37]. The study used AI denoising in order to improve image quality at lower dose levels. Currently developed AI algorithms used in radiology might be able to further reduce radiation exposure not only in PCCT but any image type in the near future. The trade-off between lower radiation dose and resource consumption for AI needs to be considered.

### 4.1. Limitations

This study was conducted on cadaveric specimens, which may not fully replicate in vivo conditions such as patient movement, tissue perfusion, and altered contrast. However, the measured navigation accuracy is consistent with previously published in vivo data, supporting the validity of the findings. The limited number of two body donors provides only little anatomical variability. However, the aim of the study was not to prove interindividual variability. It is a proof-of-concept study with the goal to assess navigation performance across different dosing regimen, but also across multiple navigation platforms. Nevertheless, repeated measurements within two specimen cannot substitute for independent replication, and the limited number of body donors remains an important limitation. Larger cadaveric and prospective in vivo studies are needed to establish generalizability across a diverse patient population.

Another limitation of the study is the small number of physicians (n = 4) performing calibration and navigation, compromising generalization of the results. This study did not evaluate registration time, which is an outcome reported by previous CT-guided imaging studies. Furthermore, quantitative image quality metrics (e.g., SNR, CNR) were not considered due to the broad availability of image quality studies in the existing literature and the different focus of the here-presented study on navigation accuracy. Notably, the requirements for image-guided navigation differ from those of diagnostic imaging, as accurate spatial registration may remain feasible even when image quality is insufficient for detailed diagnostic interpretation.

### 4.2. Future Perspectives

In the here-presented study, the minimum achievable dose was not limited by image quality, but by the PCCT scanner software: The device settings did not allow for a dose reduction to less than 5% of the baseline settings. Therefore, it was not possible to determine the threshold at which image acquisition and accurate navigation could no longer be reliably maintained. Future studies could potentially use even lower dose levels in cooperation with the respective manufacturers adjusting device settings. Imaging protocols could be developed to optimize datasets regarding their applications. Navigation-specific protocols for CT-guided navigation may require substantially less image information than conventional diagnostic examinations. Already developed and future adjusted AI denoising algorithms could be used to further optimize image usability for individual patient specific purposes [37]. Prospective in vivo studies are needed to determine whether this concept remains robust in the presence of anatomical variability, pathology, artifacts, and patient motion.

Beyond dose optimization, photon counting technology provides high spatial resolution that could be used beyond diagnostic purposes and conventional CT-guided navigation. Automated or AI-assisted segmentation could enable high-quality reconstruction of 3D models of the paranasal sinuses, skull base, orbit, and other critical neurovascular structures. These 3D models could be used for augmented or virtual reality applications, potentially facilitating preoperative planning, especially in cases of complex anatomical variations. 3D reconstructions allow for a more comprehensive display of spatial information that could improve operation planning and patient safety. It remains to be tested whether ultra-low dose PCCT imaging in combination with AI-assisted segmentation and reconstruction for skull base, paranasal sinuses or midface is feasible, which has been shown previously for non-PCCT only [38,39].

## 5. Conclusions

In this proof-of-concept study ultra-low-dose PCCT images were used for image-guided navigation of predefined endonasal and midface anatomical landmarks. Even at 5% of standard exposure, navigation accuracy was comparable to accuracy at 100% exposure. This effect was uncompromised by the use of different navigation devices. Inter-rater reliability and user satisfaction were overall high. Four different navigation systems were tested, all of which demonstrated similar results. These findings support the potential of PCCT to substantially reduce radiation burden while maintaining clinical performance. Further studies are needed to validate these results in vivo and in a larger sample size.

## Figures and Tables

**Table 1:** Photon-counting CT (PCCT) scans were acquired from two body donors (specimens 1 and 2). The corresponding Computed Tomography Dose Index (CTDIvol) and Dose Length Product (DLP), obtained at an image quality level of 24 and a tube voltage of Sn100 kV, are listed and defined as the 100% reference dose.

Specimen	1	2	Mean
mA	189	180	184.5
dose length product [mGy*cm]	50.5	60.3	55.4
CTDIvol (mGy)	3.23	3.05	3.14

**Table 2:** Mean Computed Tomography Dose Index (CTDIvol), Dose Length Product (DLP), and estimated effective radiation dose for the body donors at the reference level (100%) and at reduced dose levels of 50%, 25%, 10%, and 5% of the initial acquisition settings.

	100%	50%	25%	10%	5%
dose length product [mGy*cm]	55.4	27.5	13.8	4.5	2.2
CTDIvol (mGy)	3.14	1.55	0.78	0.26	0.13
effective radiation dose [mSv]	0.83	0.41	0.21	0.07	0.03

**Table 3:** Navigation accuracy for individual anatomical landmarks (in mm), reported as mean ± standard deviation. Measurements are presented for 100% and 5% radiation dose levels, along with the overall deviation calculated across all navigation points.

Navigation Deviation [mm]	100% Radiation Dose	5% Radiation Dose	Overall Deviation
all landmarks	2.22 ± 1.16	1.93 ± 1.05	2.01 ± 2.09
middle turbinate	1.79 ± 1.95	2.57 ± 1.97	1.85 ± 1.98
Nasopharynx	2.73 ± 2.41	3.32 ± 2.88	2.98 ± 2.58
nasion	2.04 ± 1.78	1.04 ± 0.58	1.52 ± 1.39
nasal spine	2.38 ± 2.21	1.86 ± 1.66	2.23 ± 1.94
canthus	1.86 ± 2.03	1.25 ± 1.42	1.30 ± 1.77

## Data Availability

The data presented in this study are available on request from the corresponding author due to privacy restrictions.

## Abbreviations

The following abbreviations are used in this manuscript:

ANOVA	Analysis of Variance
CT	Computed tomography
CTDI	Computed tomography dose index
DECT	Dual-energy dual-source CT
DLP	Dose length product
EID	Energy-integrating detector
ESS	Endoscopic sinus surgery
IGS	Image-guided surgery
PCCT	Photon-counting CT
SDCT	Spectral detector CT
VAS	Visual analog scale
